# Chemokine (C-X-C motif) ligand 1 is associated with tumor progression and poor prognosis in patients with colorectal cancer

**DOI:** 10.1042/BSR20180580

**Published:** 2018-07-03

**Authors:** Changhua Zhuo, Xianyi Wu, Jing Li, Dan Hu, Jinliang Jian, Changjiang Chen, Xiongwei Zheng, Chunkang Yang

**Affiliations:** 1 Department of Gastrointestinal Surgical Oncology, Fujian Cancer Hospital and Fujian Medical University Cancer Hospital, Fuzhou 350014, China; 2 Fujian Provincial Key Laboratory of Tumor Biotherapy, Fuzhou 350014, China; 3 Department of CyberKnife, Huashan Hospital, Fudan University, Shanghai 201206, China; 4 Department of Pathology, Fujian Cancer Hospital and Fujian Medical University Cancer Hospital, Fuzhou 350014, China; 5 Fujian Provincial Key Laboratory of Translational Cancer Medicine, Fuzhou 350014, China

**Keywords:** Colorectal cancer, Chemokine (C-X-C motif) ligand 1, Glycolysis, Survival analysis, Warburg effect

## Abstract

Chemokine (C-X-C motif) ligand 1 (CXCL1) is a chemotactic cytokine known to regulate cancer progression and invasion. However, the prognostic significance of CXCL1 expression in colorectal cancer (CRC) has not been fully characterized. The present study explored the clinicopathological significance and potential role of CXCL1 in the carcinogenesis and progression of CRC. The protein expression of CXCL1 was measured immunohistochemically in tissue microarrays constructed from 276 CRC patients. CXCL1 expression levels and their associations with clinicopathological characteristics and patient survival were evaluated. The effect of CXCL1 on glycolysis was also examined*.* High CXCL1 expression was detected in 165 (59.8%) cases. CXCL1 expression was correlated with tumor diameter (*P*=0.002), T stage (*P*=0.044), N stage (*P*=0.005), M stage (*P*=0.001), lymphovascular invasion (*P*=0.010), and carcinoembryonic antigen status (*P*=0.019). High CXCL1 expression was validated as an independent prognostic factor for overall survival (OS) and disease-free survival (DFS) by both univariate and multivariate Cox regression analyses (both *P*<0.05). Experimentally, expression of CXCL1 was knocked down by stable transfected short hairpin RNA, resulting in a significantly decreased rate of glycolysis both in *in vitro* assays and in patients’ samples (*P*<0.05). Silencing the expression of CXCL1 decreased the levels of the glycolytic enzymes GLUT1, HK2, and LDHA. In conclusion, by inducing glycolysis, CXCL1 plays a crucial role in both cancer progression and metastasis in CRC patients. The CXCL1 expression level is an independent prognostic factor for both OS and DFS. Moreover, CXCL1 may serve as a new biomarker and potential therapeutic target for CRC treatment.

## Introduction

Colorectal cancer (CRC) is the third most commonly diagnosed cancer and the third leading cause of cancer-related death worldwide [1]. In China, the incidence of CRC has increased in recent years as living conditions have improved and eating habits have changed [2,3]. Surgical resection remains the only curative treatment option for patients with CRC [4]. However, a considerable proportion of CRC patients develop local recurrence and distant metastasis within 5 years after surgical treatment [5,6]. The TNM staging system is widely used to predict the prognosis for CRC and to determine treatment options. However, there is considerable prognostic heterogeneity among patients with CRC of different pathologic stages, and this remains a challenge. Clinically, tumors at the same stage can lead to significantly different outcomes. Consequently, there is an urgent need to understand the molecular mechanisms underlying the progression of CRC.

Chronic inflammation is a prognostic factor for CRC [5,7]. There is a growing evidence that an inflammatory tumor microenvironment plays a critical role in the development and progression of cancer, including tumorigenesis, growth, and metastasis [8]. Chemokines compose a multifunctional family of small cytokine-like proteins that, through chemoattraction, selectively control the recruitment and migration of lymphocytes to combat infection or injury [8,9]. Recent studies have indicated that chemokines also play a vital role in tumor transformation, progression, and angiogenesis [8–10]. Chemokine (C-X-C motif) ligand 1 (CXCL1), also named GRO-α, is a member of the G protein-coupled receptor family that binds specifically to CXC chemokine receptor 2 [11,12]. CXCL1 is overexpressed in CRC and facilitates metastasis and progression [13–15]. However, the prognostic significance of CXCL1 overexpression and related underlying mechanisms are not fully understood.

In the present study, the expression of CXCL1 was investigated in 276 patients with CRC using immunohistochemical (IHC) analysis of tissue microarrays (TMAs). Additionally, *in vitro* analysis was used to classify the underlying roles of CXCL1 in CRC.

## Materials and methods

### Patient and tissue samples

Formalin-fixed, paraffin-embedded CRC tissues from 276 patients treated at Fujian Cancer Hospital between January 2012 and November 2014 were retrieved. All patients underwent radical resection and were regularly followed up. All patients were staged and pathologically graded according to the TNM staging system for CRC of the Union for International Cancer Control/American Joint Committee on Cancer (UICC/AJCC, 7th edition). The use of the clinical samples was approved by the Human Ethics Review Committee of the Fujian Cancer Hospital and Fujian Medical University Cancer Hospital, China. Written informed consent was obtained from all patients included in the study.

### Tissue microarray construction and immunohistochemical staining

The construction of TMAs from tissue samples was conducted as previously described [7]. TMAs were deparaffinized, rehydrated, and then incubated with rabbit polyclonal antibody against CXCL1 (ab86436, Abcam Inc., Cambridge, MA, U.S.A.; dilution ratio 1:50) at 4°C overnight following heat-induced epitope retrieval. Staining detection was performed using the GTVision^™^ III Kit (GK500705, Gene Tech, Shanghai, China) according to the manufacturer’s instructions. Phosphate-buffered saline was used as the negative control. A semiquantitative scoring system was used [16] with contributions from both staining intensity (0, no stain; 1+, weak stain; 2+, moderate stain; and 3+, strong stain) and the percentage of stained cells (0, <5%; 1, 5–25%; 2, 26–50%; 3, 51–75%; and 4, >75%). Scores for both the staining intensity and the percentage of positive cells were then multiplied to generate the immunoreactivity score (IS) for each case. All cases were sorted into two groups according to the IS. High expression of CXCL1 was defined as detectable immunoreactions in cytoplasm and membranes with IS ≥ 4 [17].

### Cell cultures of colon cancer cell lines

Colon cancer cell lines HCT116 and RKO were originally obtained from the American Type Culture Collection (Manassas, VA, U.S.A.). The cells were cultured in a medium according to the recommendations of the Defense Technical Information Center, supplemented with 10% fetal bovine serum (Gibco, Life Technology, Vienna, Austria) and 1% penicillin/streptomycin in a humidified 5% (v/v) atmosphere of CO_2_ at 37°C.

### Stable transfection of cell lines

Biologically active short hairpin RNAs (shRNAs) were generated using the lentiviral expression vector pLKO.1-puro. The shRNA target sequence for human CXCL1 was 5′-CGGAAAGCTTGCCTCAATCCT-3′. PLKO.1-scramble shRNA with limited homology with any known sequences in the human, mouse, and rat genomes was used as a negative control. HCT116 and RKO cells were transfected with the pLKO.1-shCXCL1 knockdown plasmid or pLKO.1-scramble. The stably transfected cells were isolated using puromycin selection to obtain stable CXCL1 knockdown cells.

### RNA isolation and quantitative real-time polymerase chain reaction

Total RNA was prepared using TRIzol® reagent (Life Technologies, Inc., Carlsbad, MD, U.S.A.), and cDNA was obtained by reverse transcription using a PrimeScript RT Reagent Kit (Cat. # RR036A, TaKaRa, Kyoto, Japan). The expression status of candidate genes and β-actin was determined by quantitative real-time PCR (RT-PCR) using an ABI 7900HT Real-Time PCR system (Applied Biosystems, U.S.A.). The primers used were human CXCL1: 5′-CCAGACCCGCCTGCTG-3′ (forward), and 5′-CCTCCTCCCTTCTGGTCAGTT-3′ (reverse); human β-actin: 5′-CTACGTCGCCCTGGACTTCGAGC-3′ (forward), and 5′-GATGGAGCCGCCGATCCACACGG-3′ (reverse). All reactions were performed in triplicate.

### Western blotting analysis

Equal amounts of cell lysates were subjected to 10% sodium dodecyl sulfate polyacrylamide gel electrophoresis (SDS-PAGE), and proteins were transferred onto polyvinylidene difluoride membranes (Bio-Rad Laboratories, Inc., Hercules, CA, U.S.A.). The membranes were probed overnight with specific primary antibodies, which were detected with corresponding secondary antibodies (Cell Signaling Technology, Danvers, MA, U.S.A.). The immunoreactive bands were visualized using enhanced chemiluminescence (Thermo Scientific, Inc., Waltham, MA, U.S.A.). The following primary antibodies were used: CXCL1 (ab86436, Abcam, Inc., Cambridge, MA, U.S.A.; dilution ratio 1:50) and β-actin (14395-1-AP, Proteintech Group, Inc., Rosemont, IL, U.S.A.). β-Actin served as the loading control.

### Glycolysis analysis

A Glucose Uptake Colorimetric Assay Kit and a Lactate Colorimetric Assay Kit (BioVision, Inc., Milpitas, CA, U.S.A.) were used to examine the glycolysis process in colon cancer cells according to the manufacturer’s protocol. RT-PCR was performed to test the expressions of glycolytic enzymes. All reactions were performed in triplicate.

### Statistical analysis

All data are shown as means ± standard deviations. Student’s *t*-test was used for statistical analysis unless otherwise noted, with *P<*0.05 considered as significant. In IHC assays of CRC samples, Spearman’s rank correlation assay was used to determine the correlation between CXCL1 and clinicopathological factors. The final follow-up was set on December 31, 2017. Survival curves were plotted using the Kaplan–Meier method and were compared using the log-rank test. The significance of various survival-related variables was assessed using the Cox regression model in multivariate analysis. Specifically, patients diagnosed at stage IV were excluded from the diseases-free survival (DFS) analysis. All statistical analyses were performed using the IBM SPSS 22.0 statistical software package (SPSS, Chicago, IL, U.S.A.).

## Results

### Clinicopathological features

Demographic, clinical, and histopathological variables are shown in Table 1. There were 166 (60.1%) male and 110 (39.9%) female patients. The median age was 57 (range, 27–85) years. The cohort comprises 24 (8.7%) cases diagnosed at stage I, 90 (32.6%) at stage II, 120 (43.5%) at stage III, and 42 (15.2%) at stage IV. The median follow-up time was 52 (range, 43–71) months.

**Table 1:** Association between CXCL1 (GRO-α) expression and clinicopathological factors in colorectal cancers

Characteristics	Total	CXCL1 expression		*P*-value
		Low expression	High expression	
Gender				0.756
Male	166	68(61.3)	98(59.4)	
Female	110	43(38.7)	67(40.6)	
Age				0.926
<60	11	68(61.3)	102(61.8)	
≥60	94	43(38.7)	63(38.2)	
Histologic grade				0.337
G0	16	5(4.5)	11(6.7)	
G1	59	20(18.0)	39(23.6)	
G2	185	77(69.4)	108(65.5)	
G3	16	9(8.1)	7(4.2)	
Tumor diameter				**0.002**
<5cm	179	84(75.7)	95(57.6)	
≥5cm	97	27(24.3)	70(42.4)	
T stage				**0.044**
T1/2	43	19(17.1)	24(14.5)	
T3	54	29(26.1)	25(15.2)	
T4	179	63(56.8)	116(70.3)	
N stage				**0.005**
N0	121	61(55.0)	60(36.4)	
N1	82	30(27.0)	52(31.5)	
N2	73	20(18.0)	53(32.1)	
M stage				**0.001**
M0	234	104(93.7)	130(78.8)	
M1	42	7(6.3)	35(21.2)	
Lymphovascular invasion				** 0.010**
Negative	187	85(76.6)	102(61.8)	
Positive	89	26(23.4)	63(38.2)	
Perineural invasion				0.570
Negative	231	95(85.6)	137(83.0)	
Positive	44	16(14.4)	28(17.0)	
CEA status				**0.019**
Normal	176	80(72.1)	96(58.2)	
High	100	31(27.9)	69(41.8)	

Abbreviation: CEA, carcinoembryonic antigen. Bold *P-*values indicate statistical significance.

### Relationship between CXCL1 expression and clinicopathological characteristics

**Figure 1:**
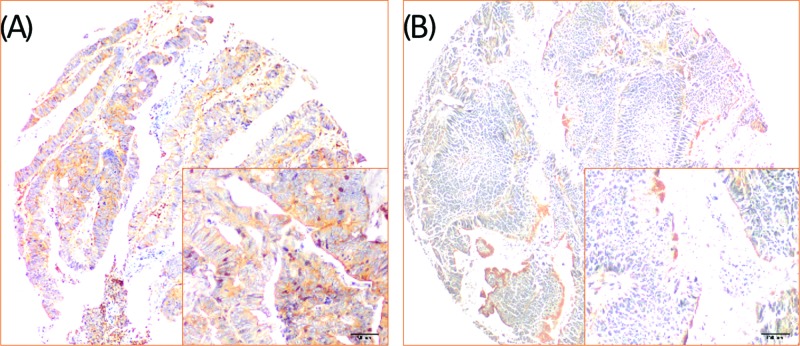
Examples of CXCL1 staining in colorectal cancer tissue samples CXCL1 exhibited membrane-accentuated expression. Representative images of high (**A**) and low (**B**) expression levels of CXCL1 in CRC tissues.

Membrane-accentuated expression of CXCL1 protein was found and was often accompanied by a cytoplasmic expression. Examples of CXCL1 staining are shown in Figure 1. High CXCL1 expression was detected in 165 (59.8%) cases, which was significantly higher than that in the normal control. CXCL1 expression and the clinicopathological characteristics are shown in Table 1. CXCL1 expression was correlated with the tumor diameter (*P=*0.002), T stage (*P=*0.044), N stage (*P=*0.005), M stage (*P=*0.001), lymphovascular invasion (*P=*0.010), and CEA status (*P=*0.019).

### Association between survival and CXCL1 expression

**Figure 2:**
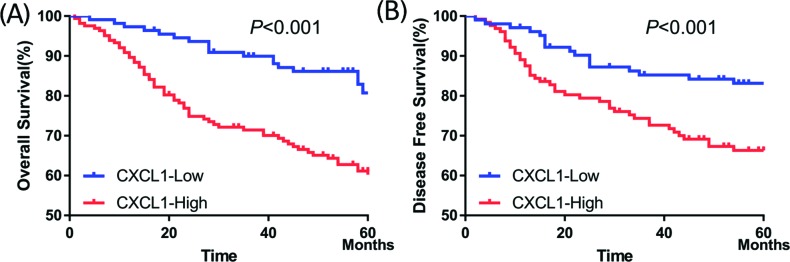
Higher CXCL1 expression correlated with worse survival in patients with CRC (**A**) Five-year overall survival rates in patients with high or low expression of CXCL1 were 61.0 and 80.7%, respectively (*X*^2^ = 14.868, *P*<0.001). (**B**) Five-year disease-free survival rates in patients with high or low CXCL1 expression were 65.7 and 82.3%, respectively (*X*^2^ = 8.441, *P=*0.004).

At a final follow-up, tumor relapse was observed in 100 (36.2%) of 276 patients, and 81 (29.3%) patients had died of the disease. CXCL1 expression was significantly associated with worse DFS (*P*<0.001; Figure 2A) and worse overall survival (OS) (*P*<0.001; Figure 2B).

The univariate Cox regression model demonstrated that the T stage (*P<*0.001), N stage (*P<*0.001), M stage (*P<*0.001), lymphovascular invasion (*P<*0.001), perineural invasion (*P=*0.043), pretreatment CEA level (*P<*0.001), and CXCL1 expression (*P<*0.001) were correlated with OS (*P<*0.001) (Table 2), whereas the T stage (*P=*0.001), N stage (*P<*0.001), lymphovascular invasion (*P=*0.002), perineural invasion (*P=*0.038), pretreatment CEA level (*P=*0.034), and CXCL1 expression (*P=*0.005) were associated with DFS (Table 3). Multivariate analysis after adjustment revealed that the T stage (*P=*0.008), M stage (*P*<0.001), and CXCL1 expression (*P=*0.013) were independent prognostic factors for OS (Table 2), whereas the T stage (*P=*0.013), N stage (*P*=0.027), and CXCL1 expression (*P=*0.037) were independent prognostic factor for DFS in CRC patients (Table 3).

**Table 2:** Univariate and multivariate survival analyses of CXCL1 expression and overall survival for patients with colorectal cancer

Variable	Univariate analysis		Multivariate analysis	
	HR (95% CI)	*P*	HR (95% CI)	*P*
Sex	0.829 (0.526–1.306)	0.418		NI
Age	1.224 (0.789–1.900)	0.367		NI
Grade	1.100 (0.786–1.541)	0.578		NI
Tumor diameter	0.706 (0.435–1.144)	0.156		NI
T stage	2.796 (1.738–4.499)	**<0.001**	1.887 (1.181–3.015)	**0.008**
N stage	1.980 (1.516–2.585)	**<0.001**	1.312 (0.955–1.803)	0.094
M Stage	9.094 (5.757–14.367)	**<0.001**	5.303 (3.047–9.228)	**<0.001**
Lymphovascular invasion	2.390 (1.545–3.697)	**<0.001**	0.990 (0.596–1.644)	0.969
Perineural invasion	1.700 (1.016–2.842)	**0.043**	1.146 (0.673–1.949)	0.616
CEA status	2.499(1.613–3.871)	**<0.001**	1.122 (0.676–1.861)	0.657
CXCL1	2.604 (1.570–4.320)	**<0.001**	1.926 (1.150–3.227)	**0.013**

NI, not included in multivariate survival analysis. Bold *P-*values indicate statistical significance.

**Table 3:** Univariate and multivariate survival analyses of CXCL1 expression and disease-free survival for patients with colorectal cancer

Variable	Univariate analysis		Multivariate analysis	
	HR (95% CI)	*P*	HR (95% CI)	*P*
Sex	0.883 (0.526–1.483)	0.639		NI
Age	1.288 (0.777–2.136)	0.326		NI
Grade	1.089 (0.740–1.604)	0.666		NI
Tumor diameter	0.716 (0.413–1.242)	0.235		NI
T stage	2.179 (1.392–3.410)	**0.001**	1.767 (1.130–2.762)	**0.013**
N stage	1.823 (1.350–2.462)	**<0.001**	1.453 (1.042–2.026)	**0.027**
Lymphovascular invasion	2.212 (1.331–3.677)	**0.002**	1.269 (0.721–2.235)	0.409
Perineural invasion	1.882 (1.036–3.420)	**0.038**	1.315 (0.711–2.433)	0.383
CEA status	1.744 (1.043–2.917)	**0.034**	1.444 (0.857–2.433)	0.167
CXCL1	2.215 (1.277–3.842)	**0.005**	1.821 (1.037–3.198)	**0.037**

NI, not included in multivariate survival analysis. Bold *P-*values indicate statistical significance.

### CXCL1 promotes glycolysis in CRC

**Figure 3:**
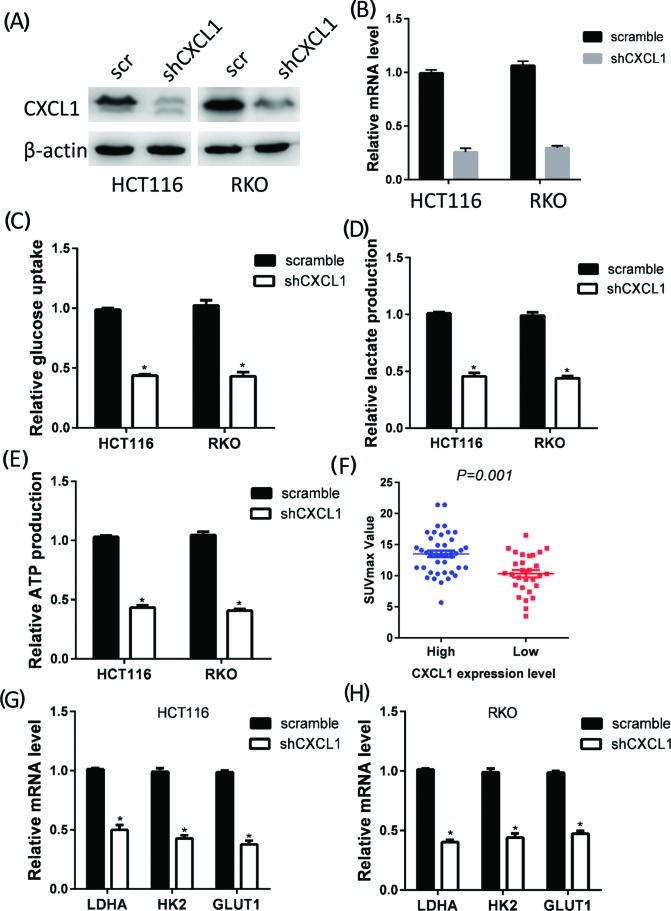
CXCL1 promotes glycolysis in patients with CRC CXCL1 expression was silenced by shRNA in HCT116 and RKO cell lines, and the knockdown efficacy was determined by Western blotting (**A**) and RT-PCR (**B**). Knockdown of CXCL1 expression significantly decreased glucose consumption (**C**), lactate production (**D**), and ATP production (**E**). (**F**) Patients with high CXCL1 expression exhibited higher SUV_max_ than those with low CXCL1 expression (*P=*0.001). (**G** and **H**) Silencing CXCL1 expression decreased GLUT1, HK2, and LDHA mRNA expression in HCT116 (G) and RKO (H) cell; *P<0.05.

Tumor growth and metastasis require that glucose metabolism be reprogrammed to glycolysis. Because CXCL1 expression was correlated with larger tumor sizes and advanced tumor stages, we further investigated the effect of CXCL1 expression on CRC glycolysis. CXCL1 expression was knocked down in HCT116 and RKO cells by shRNA. The efficiency of the knockdown was verified by RT-PCR and Western blotting (Figure 3A,B). We then calculated glucose utilization, lactate concentrations, and ATP production in CXCL1 knockdown cells. The CXCL1 expression knockdown strongly decreased glucose utilization, lactate concentrations, and ATP production in HCT116 and RKO cells (Figure 3C–E). The maximum standardized uptake value (SUV_max_) in positron emission tomography–computed tomography scans is a reflection of the Warburg effect activity. In a cohort of 72 patients, higher SUV_max_ were observed in patients with higher levels of CXCL1 expression (Figure 3F). Glycolysis is a multistep enzymatic reaction involving a series of rate-limiting enzymes. Silencing the expression of CXCL1 up-regulated or down-regulated several rate-limiting enzymes in the glycolysis process, most significant of which were GLUT1, HK2, and LDHA (Figure 3G,H).

## Discussion

Metastasis and recurrence occur in approximately 50% of CRC patients after radical resection and are the major causes of CRC-related deaths [18]. Studies of prognosis and of prognostic factors to predict the risk of recurrence and metastasis for individual CRC patients are ongoing, and could affect clinical practice. Established biomarkers such as KRAS, BRAF, and microsatellite instability status play critical roles in predicting prognosis and in the selection of patients suitable for personalized therapy [19]. Evidently, much research has focused on improving patient care and understanding the biology of CRC.

The chemokine family is receiving increased attention as multifunctional proteins that regulate many cellular phenotypes in addition to their classical roles as chemotactic molecules [8]. In the context of tumor biology, specific chemokines are angiogenic or antistatic [20,21]. They regulate survival, cell cycle progression, growth, and cell–cell interaction [10,14,22,23]. In the present study, we investigated CXCL1 expression in CRC by IHC analysis of TMAs and found that CXCL1 expression was significantly higher in cancer tissues than in adjacent normal tissues. High CXCL1 expression is correlated with larger tumor size and advanced tumor stage. Importantly, CXCL1 expression was an independent prognostic biomarker for CRC patients.

Uncontrolled proliferation is one of the crucial features of malignant tumors. It has been proposed that reprogramming energy metabolism is critical to maintain such behavior, and is a hallmark of cancer cells [24]. The Warburg effect, also known as aerobic glycolysis, has been intensively studied in recent decades [25]. The Warburg effect not only provides ATPs and nutrients to rapidly growing cancer cells, but also creates an acidic environment that leads to the destruction of extracellular matrix, thereby facilitating metastasis. Because CXCL1 is positively correlated with tumor diameter and advanced tumor stage, we hypothesize that CXCL1 may regulate glycolysis in CRC. As anticipated, silencing CXCL1 expression significantly decreased the glycolysis rate in CRC cell lines. The metabolic reprogramming induced by CXCL1 may be required for cancer cell growth and metastasis.

There were some limitations in our study. First, no *in vivo* experiments using animals were performed to observe the effects of CXCL1 on progression and metastasis. Second, although we demonstrated that CXCL1 promoted glycolysis in CRC and up-regulated several glycolytic enzymes, further study is needed to classify how CXCL1 induces the glycolytic process.

In conclusion, we have provided firm evidence that by inducing glycolysis, CXCL1 plays a crucial role in CRC progression and metastasis. The CXCL1 expression level was an independent adverse prognostic factor for both OS and DFS. Moreover, CXCL1 may serve as a new biomarker and potential therapeutic target for CRC treatment.

## Abbreviations

CEA: carcinoembryonic antigen
CRC: colorectal cancer
CXCL1: chemokine ligand 1
DFS: diseases-free survival
GLUT1: glucose transporter 1
GRO-α: growth-related oncogene-alpha
HK2: hexokinase 2
HR: hazard ratio
IHC: immunohistochemical
IS: immunoreactivity score
LDHA: lactate dehydrogenase
OS: overall survival
RT-PCR: real-time PCR
shRNA: short hairpin RNA
SUV_max_: maximum standardized uptake value
TMA: tissue microarrays
TNM: tumor node metastasis
